# Best-bet integrated strategies for containing drug-resistant trypanosomes in cattle

**DOI:** 10.1186/1756-3305-5-164

**Published:** 2012-08-08

**Authors:** Erick O Mungube, Oumar Diall, Maximilian PO Baumann, Antje Hoppenheit, Barbara Hinney, Burkhard Bauer, Yousouf Sanogo, Brehima Maiga, Karl-Hans Zessin, Thomas F Randolph, Peter-Henning Clausen

**Affiliations:** 1 Institute for Parasitology and Tropical Veterinary Medicine, Freie Universität Berlin, Königsweg 67, D-14163, Berlin, Germany; 2 Kenya Agricultural Research Institute, Katumani Research Centre, P.O. Box 340–90100, Machakos, Kenya; 3 FAO Regional Office, Accra Ghana, P.O. Box GP 1628, Accra, Ghana; 4 International Animal Health, Freie Universität Berlin, Königsweg 67, D-14163, Berlin, Germany; 5 Laboratoire Central Vétérinaire (LCV), BP 2295, Bamako, Mali; 6 Pan African Tsetse and Typanosomosis Eradication Programme (PATTEC) Mali, BP 9125, Bamako, Mali; 7 International Livestock Research Institute (ILRI), P.O. Box 30709, Nairobi, 00100, Kenya

**Keywords:** Trypanosomosis, Trypanocidal drug resistance, Cattle, Tsetse control, Helminth control, Mali

## Abstract

**Background:**

African animal trypanosomosis is a major constraint to the rearing of productive livestock in the sub-humid Sudan-Sahel zone of West Africa where cotton is grown. Trypanosomosis is mainly controlled using trypanocidal drugs, but the effective use of drugs is threatened by the development of widespread resistance. This study tested integrated best-bet strategies for containment and/ or reversal of trypanocide resistance in villages in south-east Mali where resistance has been reported.

**Methods:**

Four sentinel villages each from an intervention area (along the road from Mali to Burkina Faso) and a control area (along the road from Mali to Côte d’Ivoire) were selected for the study. Tsetse control was based on deltamethrin-treated stationary attractive devices and targeted cattle spraying between March 2008 and November 2009. Trypanosome-positive cattle were selectively treated with 3.5 mg/kg diminazene aceturate. Strategic helminth control using 10 mg/kg albendazole was also undertaken. During the intervention, tsetse densities along drainage lines, trypanosome infections and faecal egg counts in risk cattle (3 to 12 months of age) were monitored.

**Results:**

Catch reductions of 66.5 % in *Glossina palpalis gambiensis* and 90 % in *G. tachinoides* were observed in the intervention area. Trypanosome prevalence was significantly (p < 0.05) lower in the intervention area (2.3 %; 1.3-3.6 %) compared to the control area (17.3 %; 14.8-20.1 %). Albendazole treatment resulted in a faecal egg count reduction of 55.6 % and reduced trypanosome infection risk (2.9 times lower than in the placebo group) although not significantly (p > 0.05). Further studies are required before confirming the existence of albendazole resistant strongyles in the study area.

**Conclusion:**

Integration of best-bet strategies in areas of multiple drug-resistance is expected to reduce trypanosome infection risk thus contributing to containment of trypanocidal drug resistance. Integrated best-bet strategies could therefore be considered a viable trypanosomosis control option especially in areas where multiple drug-resistance has been reported.

## Background

In the region of Sikasso, Mali – as in other parts of the sub-humid Sudan-Sahel zone of West Africa where cotton is grown - African animal trypanosomosis (AAT) is a major obstacle to the promotion of productive and sustainable animal husbandry management systems [1]. The high trypanosomosis risk in this area has led to increased use of trypanocides to maintain trypano-susceptible zebu cattle kept for animal traction and production [2] resulting in trypanocide resistance which was first detected and reported in Burkina Faso [3-6], and later in south-east Mali [7-9].

In sub-Saharan Africa, about 35–70 million drug doses are annually used for the control of trypanosomosis [10]. Their efficacy for controlling this disease is however severely compromised by resistance, yet new molecules may not be available any time soon. Strategies to prolong the efficacy of existing trypanocidal drugs are therefore required to allow sustainable livestock production in the high risk areas.

Little is known about containing or reversing trypanocide resistance, in contrast to knowledge of how to deal with antibiotic resistance. Questions remain about the mechanisms of resistance (single or multiple), genesis of resistance (uni- or multifocal), spread of resistance to new areas (role of vectors and cattle movement) and persistence of resistance. Since it is assumed that cyclically transmitted trypanosomes in Africa cannot persist in the absence of tsetse, vector eradication could be an effective means of eliminating resistance. Methods for control or suppression of localized tsetse populations have been developed and have repeatedly been shown to be highly effective [11]. Community-based bait methods using insecticide-treated cattle and traps [12,13] are particularly attractive. Eradication of tsetse flies from the continent at present appears to be a goal, unattainable in the near future unless considerable investment is made. Despite the effectiveness of vector control in controlling AAT, it cannot, on its own, completely eliminate resistant trypanosome populations from an area. There is a continuous risk of spreading the residual resistant trypanosomes in the event of tsetse reinvasion. Combining vector control with other health enhancing packages such as good nutritional practices or control of co-infections could improve control of resistant trypanosomes and thereby prolong the use of trypanocides. Supplementation with proteins together with treatment of co-infections, particularly those causing immunosuppression such as *Haemonchus contortus*, bolsters immunological competence and might help cattle to self-cure from resistant trypanosomes [14].

This study with the objective of containing or reversing trypanocide resistance tested best-bet integrated trypanosomosis control strategies. An integrated package of vector control, strategic helminth control and targeted diminazene treatments was implemented and evaluated in south-east Mali where multiple-drug resistant *T. congolense* had been previously detected [9]. The paper describes and compares the evolution of tsetse densities, trypanosome infections and faecal egg counts in risk group cattle during and after testing.

## Methods

### Study area description

**Figure 1:**
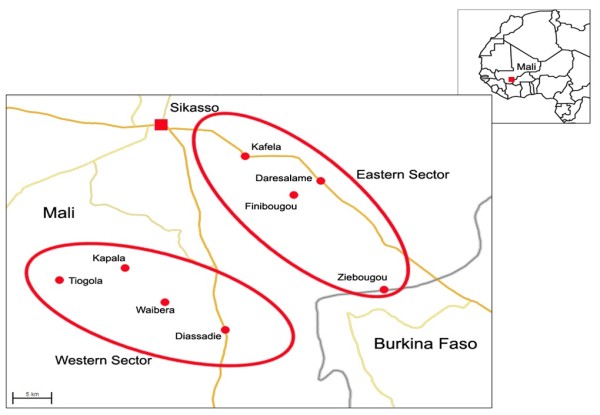
Study map showing the study sites (circled) and study villages (dotted red), south-east Mali.

The study was conducted in the administrative district of Sikasso in south-east Mali. Sikasso lies on 11° 19’ N and 5° 40’ W at an altitude of 410 m (1348 feet) above sea level (Figure 1). Two areas were selected: an eastern sector located along the Mali-Burkina Faso road where tsetse control was implemented (intervention area) and a western sector along the Mali-Côte d’Ivoire road without tsetse control (control area). Within both areas, four villages were identified as study sites. The two areas were comparable ecologically and in terms of their agricultural production system [15].

### Cattle population (reference population)

The reference population consisted of herds of cattle, both trypanosusceptible zebu and trypanotolerant breeds of any sex and age. Preference was given to herds whose animals had participated in the pre-intervention survey [9].

### Trypanosomosis risk and drug resistance before intervention

Pre-intervention trypanosome risk and trypanocidal drug resistance were comparable between the intervention and control areas [9]. Of the 796 sampled cattle from the 8 study villages, 125 (15.7 %) were trypanosome-positive based on dark-ground phase microscopy (BCT). Although trypanosome prevalence was slightly higher in the villages of the control area (17.5 %) compared to those in the intervention area (13.9 %) there was no significant (p > 0.05) difference between the sites. *Trypanosoma congolense* was the dominant trypanosome species in both areas accounting for 73 % (91/125). Trypanosome-positive cattle from each village were randomly allocated into groups treated with isometamidium chloride (ISMM) (0.5 mg/kg bw, Trypamidium®, Merial, France) or diminazene aceturate (DIM) (3.5 mg/kg bw, Veriben®, Ceva Animal Health Inc., France) [9]. The treated cattle were monitored for trypanosomes on days 14 and 28 post-treatment. Multiple drug resistant *T. congolense* strains and ISMM resistant *T. vivax* were detected in both intervention and control area [9]. Twenty (31.7 %) of the 63 cattle on ISMM treatment still had trypanosomes 14 days post-treatment. Of the 43 aparasitaemic cattle monitored to day 28, 25.6 % (11) became parasitaemic resulting in a cumulative ISMM failure rate of 49.2 % (31/63). *Trypanosoma congolense* accounted for 77.4 % (24/31) of failed ISMM treatments (the remaining treatment failures were due to *T. vivax*). Of the 62 cattle treated with DIM 30.6 % (19/62) could not be cured. DIM treatment did not cure 42.2 % (19/45) of *T. congolense* infections whereas all *T. vivax* positive cattle were still successfully treated.

### Study design

The study was conducted in two phases: an intervention phase (March 2008 to October 2009) and a post-intervention phase (November 2009).

### Intervention phase

### Study sample (risk group cattle)

The study sample comprised cattle between 3 to 12 months of age (risk group cattle). Study cattle were firstly recruited from the reference population by means of a cattle census conducted between April and May 2008 in the 8 study villages (4 from the intervention area and 4 from the control area). Risk group cattle were ear-tagged and enumerated by herd and village. Upon recruitment, the risk group cattle were retained as study subjects throughout the study period. Calves born from participating herds during the study period also entered the study sample.

### Tsetse control

Tsetse control took place in 16 villages, including the 4 study villages of the intervention area from March 2008 to November 2009 over approximately 500 km^2^, using deltamethrin treated stationary attractive devices (SADs) during the dry season and targeted deltamethrin spraying of cattle during the rainy season. A total of 957 locally tailored targets consisting of blue cloth (35 % cotton and 65 % polyester) measuring 50 cm x 100 cm and black bands measuring 25 cm x 100 cm on either side were used. Each target (1 m^2^) was impregnated with 0.025 % deltamethrin (DECIS®, Roussel-Uclaf, France) amounting to 300 mg of deltamethrin on 1 m^2^ of target. Likewise, 401 monoconical Vavoua traps [16], each measuring 3 m^2^ impregnated with 900 mg of deltamethrin (Glossinex®, AgrEvo, Zimbabwe) were used. The impregnated SADs were deployed 100–300 m apart along drainage lines or at 30 m apart in points of frequent contact between tsetse and its hosts (man and livestock).

The SADs were withdrawn at the start of the rainy season (June 2008) and bi-weekly targeted cattle spraying (limbs, lower abdominal area, thoracic and brisket regions) using 0.05 % deltamethrin (Butox®, Intervet International, the Netherlands) commenced. An estimated 4000 cattle from all 16 villages of the intervention area were sprayed during this campaign. In December 2008, the SADs were re-impregnated and re-deployed and again withdrawn in June 2009 at the onset of the rains when targeted cattle spraying resumed.

### Selective treatment with diminazene aceturate (DIM)

Selective DIM treatments were administered to trypanosome-positive cattle or to trypanosome-negative cattle with PCV of < =20 %. These treatments using Veriben® (Ceva Animal Health Inc., France) at 3.5 mg/kg body weight (b.w.) were given to the risk group cattle in both areas at every monitoring visit.

### Strategic helminth control

All villages from the intervention area and the villages of Diassadie and Waibera from the control area were part of the strategic helminth control trial. Risk group cattle allocated to albendazole treatment were drenched *per os* using 10 % albendazole (10 mg/kg b.w. Albenzole®, Kela Laboratories, Belgium) in June 2008, November 2008, June 2009 and November 2009 (beginning and end of the rainy season). Those belonging to the control were simultaneously treated with reconstituted milk powder (placebo).

### Monitoring

### Tsetse flies

One pre-intervention survey was conducted in November 2007. Five unbaited and untreated bi-conical traps [17] per village were deployed at intervals of about 100–200 m in the forest galleries along the drainage lines. During the intervention phase, 4 surveys were conducted in June 2008, November 2008, February 2009 and June 2009 to monitor changes in tsetse catches following tsetse control. Five traps per village were deployed in areas where contact between tsetse and humans or their cattle were expected, giving a total of twenty traps per area per monitoring visit. The traps remained in the forest galleries for 24 hours, after which the captured flies were counted and separated according to species and sex.

### Trypanosome prevalences

Five surveys took place between June 2008 and November 2009. June 2008 and June 2009 represented the onset of the rainy season while November 2008 and November 2009 corresponded to the end of the rainy season; the February 2009 survey took place in the middle of the dry season. During the monitoring surveys, jugular blood samples were collected from the risk group cattle in vacutainer tubes containing di-sodium salt of ethylene diamine tetra-acetate (EDTA) and examined for packed cell volumes (PCV) and trypanosomes using the dark-ground phase microscopy [18].

### Faecal egg counts (FECs)

Faecal samples from risk group cattle were examined for helminth eggs in November 2008, February 2009, June 2009 and November 2009. Faeces were rectally collected and labelled with animal tag number, breed, sex, date and herd identity. The modified McMaster technique was used for quantifying the faecal egg counts [19].

### End of intervention

### Tsetse density

At the end of the intervention, a final survey was conducted in November 2009 using a similar methodology as described for the tsetse fly monitoring surveys above.

### Trypanosome prevalence

A concluding survey was also undertaken in November 2009 in both areas. A systematic sampling approach as described by Dohoo *et al.*[20] was used to select study cattle from the reference population. It was estimated that each of the two areas had about 800 cattle of which a sample of 400 cattle was required. This gave a sampling proportion of 0.5, hence, using systematic sampling, the first animal was randomly selected and thereafter every second animal.

### Faecal egg counts and faecal egg count reduction test (FECRT)

FECRT was undertaken in November 2009 to assess the efficacy of albendazole against strongyles. Faecal egg counts of albendazole treated cattle at days 0 and 14 post-treatment were compared to those of control (placebo) cattle in accordance with the method of Coles *et al.*[21]. Half of the risk group cattle allocated to albendazole treatment had been treated with albendazole (Albenzole® 10 % suspension, Kela laboratories, Belgium) from Malian markets and the other half with German albendazole (Albendazol® 10 % suspension, aniMedica, Südfeld, Germany). The control group was simultaneously treated with a placebo.

### Data analysis

Intervention and post-intervention phase tsetse catches and trypanosome infections were recorded. Since a total of 20 traps were used in each area, flies/trap per day (FTD) for every monitoring was calculated by dividing total fly catch by 20 traps. Reduction in FTD was calculated by comparing the November 2007 FTD (pre-intervention) with that of November 2009 (post-intervention catches). FTD comparisons between areas were done using Kruskal-Wallis test. Pearson chi-square (χ^2^) was applied to test differences in trypanosome prevalence across study areas. Student *t*-test differentiated PCVs of study cattle whereas Mann–Whitney test differentiated mean FECs for the albendazole and control group cattle. Incidence density rates (IDR) for albendazole and placebo cohorts were calculated as the number of trypanosome infections between monitoring visits over the cattle-months at risk over the same period. Risk time for individual risk group cattle was estimated by the difference between the calendar date for the preceding and current survey; individual risk times were then summed for herds, villages and aggregated to the area level. FECRT was estimated using the method of Coles *et al.*[21]. Analysis was conducted in SPSS version 18 and the online program OpenEpi (http://www.openepi.com) used for calculating confidence intervals.

### Ethical clearance

After initial instructions all treatments with ectoparasiticides were part of the responsibility of local village committees. All anthelmintic and trypanocidal treatments were performed in full compliance with national veterinary regulations and upon agreement by the local village authorities.

## Results

### Tsetse catches

Only *Glossina palpalis gambiensis* and *G. tachinoides* were caught with the former dominating catches (Table 1). Before intervention, FTDs were not significantly (p > 0.05) different between the two study areas although FTDs were slightly higher in the intervention area. Tsetse control significantly (p < 0.05) reduced FTD in the intervention compared to the control area. Over the monitoring period, *G. p. gambiensis* catches were reduced by 66.5 % (from FTD of 8.35 in November 2007 to a mean FTD of 2.8 in November 2009). At the same time *G. tachinoides* catches dropped by 90 % (from FTD of 4.50 in November 2007 to a mean FTD of 0.45 in November 2009). An increase of *G. p. gambiensis* catches by 95.2 % was recorded in the control area (from FTD of 6.45 in November 2007 to a mean FTD of 12.59 in November 2009) while catches of *G. tachinoides* decreased by 31 % (from FTD 4.2 in November 2007 to a mean FTD of 2.9 in November 2009) over the same period. In the intervention area, fly catches fluctuated over time with February 2009 and June (2008 and 2009) catches being lower than those of November (2008 and 2009). Fluctuations in tsetse density were also observed in the control area with June 2008 recording the lowest catches of both tsetse species. A gradual increase in catches was noted in November 2008 and February 2009 before declining again in June 2009. Catches of both tsetse species increased in November 2009.

**Table 1:** Tsetse fly catches by area and monitoring phase during the pre-intervention, intervention and post-intervention phase in south-east Mali (November 2007 to November 2009)

**Monitoring dates**	**Intervention area**						**Control area**					
	***G. p. gambiensis***^*2*^		***G. tachinoides***^*3*^		**Total**		***G. p. gambiensis***^*2*^		***G. tachinoides***		**Total**	
	**Catch**	**FTD**^4^	**Catch**	**FTD**^3^	**Catch**	**FTD**^4^	**Catch**	**FTD**^3^	**Catch**	**FTD**^4^	**Catch**	**FTD**^4^
**Pre-Intervention**^**1**^												
Nov. 2007	167	8.35	91	4.5	258	12.9^a^	129	6.45	84	4.2	213	10.65^a^
**Intervention**												
Jun. 2008	0	0	0	0	0	0^b^	40	2	25	1.25	65	3.25^a,c^
Nov. 2008	11	0.55	3	0.15	14	0.70^b^	73	3.65	60	3	133	6.65^a^
Feb. 2009	4	0.2	0	0	4	0.2^b^	93	4.65	70	3.5	163	8.15^a^
Jun. 2009	2	0.10	0	0	2	0.1^b^	80	4	51	2.55	131	6.55^a^
**Post-Intervention**												
Nov. 2009	56	2.8	9	0.45	65	3.25^b^	259	12.59	58	2.9	317	15.85^a^
**Cumulative area total**	73	0.73	12	0.12	85	0.85	545	5.45	264	2.64	809	8.09

^1^Tsetse catches for the pre-intervention phase [9,15].

^*2*^ *G. p. gambiensis* = *Glossina palpalis gambiensis*.

^*3*^ *G. tachinoides* = *Glossina tachinoides*.

^4^FTD = Flies per trap per day; FTD values with different letter superscripts are significantly (Kruskal-Wallis test; p < 0.05) different along the row and column of comparison.

### Trypanosome prevalence during intervention

Trypanosome prevalence dropped significantly (p < 0.05) in the intervention area after the start of tsetse control, ranging between 0 % and 7.1 % compared to the control area with 14.1 % to 20.4 % (Table 2). Seasonal fluctuations were evident with highest prevalences occurring during November (end of rainy season) and lowest during February 2009 (mid dry season). *Trypanosoma congolense* and *T. vivax* were detected in both areas, with *T. vivax* being the dominant trypanosome species. No *T. brucei* were detected.

**Table 2:** Trypanosome prevalences in risk group calves by area and monitoring dates during the intervention (June 2008-June 2009) and post-intervention phase (November 2009) in south-east Mali

**Monitoring dates**	**Intervention area**							**Control area**						
	**Trypanosome positive cattle**				**No. cattle**	**Prev %**^4^	**95 % CI**^5^	**Trypanosome positive cattle**				**No. cattle**	**Prev %**^4^	**95 % CI**^5^
	**T.c.**^1^	**T.v.**^2^	**Mixed**^3^	**Total**				**T.c.**^1^	**T.v.**^2^	**Mixed**^3^	**Total**			
Jun. 2008	1	3	0	4	71	5.6^a^	1.8-13.0	2	14	0	19	84	19.0^a^	11.7-28.5
Nov. 2008	2	7	0	9	126	7.1^a^	3.5-12.7	13	17	2	32	157	20.4^a,b^	14.6-27.2
Feb. 2009	0	1	0	1	139	0.7^b,c^	0.0-3.5	11	10	1	22	163	14.1^a^	9.4-20.1
Jun. 2009	1	1	0	2	161	1.2^b,c^	0.2-4.0	11	13	2	26	175	16.0^a^	10.2-20.7
Nov. 2009	0	0	0	0	212	0^b^	0	21	15	1	37	188	20.2^a^	14.9-26.4
**Total/Mean**	**4**	**12**	**0**	**16**	**709**	**2.3**^b,c^	**1.3-3.6**	**58**	**69**	**6**	**133**	**767**	**17.3**^a^	**14.8-20.1**

^1^ T.c. = *Trypanosoma congolense*.

^2^ T.v. = *T. vivax*.

^3^Mixed trypanosome infection were *T. congolense* and *T. vivax*.

^4^Prev. % = Percent prevalence.

^5^95 % CI = 95 % confidence interval.

Trypanosome prevalences with different letter superscripts are significantly (Pearson *χ*^2^ test; p < 0.05) different from those of Nov 2007 along the row and/ or column of comparison.

### Strongyle faecal egg counts (FECs)

In the intervention area, the FEC of albendazole-treated risk group cattle ranged between 0–2500 and that of placebo treated cattle between 0–4700. In the control area, FECs for albendazole-treated cattle ranged between 0–900 and that of placebo-treated cattle between 0–1100. Although control (placebo) group risk cattle had slightly higher FECs than albendazole treated cattle, this was not statistically (p > 0.05) different (Table 3).

**Table 3:** Descriptive statistics of trichostrongyle (mean ± standard deviation) faecal egg counts (FECs) in risk group cattle in south-east Mali (November 2008 to November 2009)

**Village**	**November 2008**		**February 2009**		**June 2009**		**November 2009**	
	**Albendazole**	**Placebo**	**Albendazole**	**Placebo**	**Albendazole**	**Placebo**	**Albendazole**	**Placebo**
**Intervention area**	**n = 50**	**n = 61**	**n = 61**	**n = 66**	**n = 66**	**n = 75**	**n = 84**	**n = 82**
Kafela	144 ± 147	359 ± 301	8 ± 19	130 ±148	183 ± 234	255 ± 549	865 ± 586	708 ± 587
Finibougou	150 ± 120	269 ± 258	10 ± 28	48 ± 66	98 ± 173	182 ± 238	500 ± 293	810 ± 601
Daresalame	254 ± 352	127 ± 122	25 ± 88	57 ± 70	158 ± 124	237 ± 252	426 ± 289	695 ± 1072
Ziébougou	458 ± 618	300 ± 292	21 ± 50	90 ± 84	146 ±121	300 ± 371	938 ± 637	1027 ± 1051
**Area total**	**212 ± 301**	**276 ± 268**	**16 ± 52**	**83 ± 108**	**144 ± 322**	**232 ± 265**	**668 ± 505**	**787 ± 807**
**Control area**	**n = 13**	**n = 12**	**n = 17**	**n = 17**	**n = 11**	**n = 14**	**n = 11**	**n = 18**
Diassadié	130 ± 134	133 ± 83	9 ± 20	79 ± 92	138 ± 212	68 ± 108	341 ± 284	280 ± 286
Waibera	17 ± 29	33 ± 58	0	30 ± 45	17 ± 29	17 ± 29	0	617 ± 562
**Area total**	**104 ± 127**	**108 ± 87**	**9 ± 20**	**65 ± 82**	**105 ± 186**	**57 ± 98**	**341 ± 284**	**336 ± 248**

n = Number of risk cattle that were faecal sampled.

Other helminth eggs including eggs of *Strongyloides*, *Toxocara, Capillaria,Trichuris* and *Moniezia* species were also detected but their numbers were too low for further statistical analysis. Eggs of *Strongyloides* species and *Toxocara* species occurred only in risk cattle aged <12 months.

### Effect of strategic treatment of albendazole on trypanosome infection risk

Albendazole-treated risk group cattle in the intervention area had a lower trypanosome incidence density rate (IDR) compared to the placebo group (Table 4). However, this difference was not significant (p > 0.05). The rate ratio (RR) between these two treatment groups was 2.889 (95 % CI: 0.782-10.67). In the control area, the difference in risk between albendazole treated cattle and those belonging to the placebo group was small and also not significantly (p > 0.05) different.

**Table 4:** Trypanosome incidence density rates (IDR) for risk group cattle treated with albendazole and placebo treatment within the intervention and control areas of south-east Mali (June 2008 to November 2009)

**Monitoring dates**	**Albendazole treated risk cattle cohort**				**Placebo treated risk cattle cohort**			
	**Trypanosome cases**	**Cattle-months**	**IDR**	**95 % CI**^5^	**Trypanosome cases**	**Cattle-months**	**IDR**	**95 % CI**^5^
**Intervention area**								
Jun - Nov 2008	1	247.6	0.004	0.000-0.020	8	293.6	0.031	0.010-0.050
Nov 2008 – Feb 2009	1	192.8	0.005	0.000-0.026	0	209.2	0	0
Feb – Jun 2009	1	240.3	0.004	0.000-0.021	1	256.3	0.004	0.000-0.019
Jun – Nov 2009	0	517.5	0	0	0	493.1	0	0
**Total**	**3**^**1**^	**1198.2**	**0.003**	**0.001-0.007**	**9**^**2**^	**1252.2**	**0.007**	**0.004-0.013**
**Control area**								
Jun - Nov 2008	3	56.7	0.053	0.013-0.144	4	65.2	0.061	0.019-0.148
Nov 2008 – Feb 2009	3	52.8	0.057	0.014-0.155	2	48.6	0.041	0.007-0.136
Feb – Jun 2009	7	45.2	0.155	0.068-0.306	5	49.7	0.10	0.037-0.223
Jun – Nov 2009	2	73.8	0.027	0.005-0.090	2	108.2	0.002	0.003-0.061
**Total**	**15**^**3**^	**228.5**	**0.066**	**0.038-0.106**	**13**^**4**^	**272.3**	**0.048**	**0.027-0.080**

^1^ Two *T. vivax* and one *T. congolense*.

^2^ Seven *T. vivax* and two *T. congolense*.

^3^ Six *T. vivax*, eight *T. congolense* and one mixed infection (*T. congolense* and *T. vivax*).

^4^ Five *T. vivax,* six *T. congolense* and two mixed infections (*T. congolense* and *T. vivax*).

^5^95 % CI = 95 % confidence interval.

### Post-intervention trypanosome prevalence

Before intervention (November 2007), trypanosome prevalences in both areas were not significantly (p > 0.05) different (Table 5). After intervention, there was a significant drop (p < 0.001) in the intervention area from 13.9 % before to 0.8 % post-intervention. A drop in prevalence from 17.5 % to 5.8 % was also observed in the control area over the same period. *Trypanosoma congolense* and *T. vivax* were the only trypanosomes identified, though no *T. congolense* were detected in the intervention area. In the control area, *T. congolense* still dominated, accounting for 76 % of all infections, as before intervention. No *T. brucei* or mixed trypanosome infections were diagnosed.

**Table 5:** **Trypanosome prevalences in cattle before intervention (November 2007)**[9]**and after intervention (November 2009) by area in south-east Mali**

**Monitoring dates**	**Intervention area**							**Control area**						
	**Trypanosome positive cattle**				**No. cattle**	**Prev %**^**4**^	**95 % CI**^**5**^	**Trypanosome positive cattle**				**No. cattle**	**Prev %**^**4**^	**95 % CI**^**5**^
	**T.c.**^**1**^	**T.v.**^**2**^	**Mixed**^**3**^	**Total**				**T.c.**^**1**^	**T.v.**^**2**^	**Mixed**^**3**^	**Total**			
Nov. 2007	41	14	0	55	396	13.9^a^	10.7-17.6	50	20	0	70	400	17.5 ^a^	14.0-21.5
Nov. 2009	0	3	0	3	393	0.8^b^	0.2-2.1	16	5	0	21	362	5.8 ^b^	3.7-8.6

^1^ *T.c.* = *Trypanosoma congolense*.

^2^ *T.v.* = *T. vivax*.

^3^Mixed trypanosome infection were *T. congolense* and *T. vivax*

^4^Prev. % = Percent prevalence; Trypanosome prevalences with different letter superscripts are significantly (Pearson *χ*^2^ test; p < 0.05) different from those of Nov 2007 along the row and/ or column of comparison.

^5^95 % *CI* = 95 % confidence interval.

### Strongyle faecal egg count reduction (FECR) test

Albenzole®, Kela, Belgium resulted in a FECR of 55.6 % (95 % CI: 46.7-64.0 %) compared to 79.3 % (95 % CI: 71.9-85.7 %) for Albendazol®, aniMedica, Südfeld, Germany (Table 6). Although the threshold of 90 % FECR was not attained for both drugs, Albendazol® still had significantly (p < 0.05) higher efficacy than Albenzole®.

**Table 6:** **Results of the trichostrongyle faecal egg count reduction test (FECRT)**[21]**of risk group cattle treated with albendazole (10 mg/kg bw) or placebo in south-east Mali (November 2009)**

	**Placebo**	**Treatment with**	
		**Albenzole®**^2^	**Albendazol®**^3^
Number sampled (n)	84	43	41
Mean pre-treatment EPG^1^	773	685	651
Mean post-treatment EPG^1^	493	219	102
% Reduction	36.2	55.6	79.3
Lower 95 % CL	32.9	46.7	71.9
Upper 95 % CL	39.7	64.0	85.7

^1^*EPG* = Eggs per gram faeces.

^2^Albenzole®, Kela, Belgium (used for carrying out the strategic helminth control scheme).

^3^Albendazol®, aniMedica, Südfeld, Germany.

## Discussion

Tsetse densities in the intervention and control sites were not significantly different before intervention. The control measures significantly reduced catches of both *Glossina palpalis gambiensis* and *G. tachinoides*. The percent FTD reduction in catches of *G. p. gambiensis* in this study was lower than that reported for the same tsetse species in neighbouring Burkina Faso when deltamethrin pour-on was used [22]. Spray treatments of cattle with 0.05 % deltamethrin have a lower persistency than pour-on treatments with 1 % (0.75 %) of deltamethrin. The number of treated cattle may have been insufficient to achieve a higher reduction of *G. p. gambiensis*. Another hypothesis may be that a reinvasion of this species from neighbouring untreated areas occured. Catches of both tsetse species were significantly reduced between June 2008 and June 2009 and, FTDs of *G. tachinoides* reached 0. Although fly catches in the control area remained high, they nevertheless showed a gradual decline. The close proximity of two areas (approximately 35 km apart) could have allowed a spill-over effect with a possibility that cattle in the control area were also treated with insecticides by farmers, hence the decline in the tsetse catch. In the control area, the tsetse catch increased from June 2008 (end of the rainy season) to February 2009 (middle of the dry season) before declining again in June 2009 (start of the rainy season). Riverine tsetse species may disperse away from drainage lines when relative humidity (RH) rises (wet season) and during the dry season retreat to drainage lines, which have a micro-climate able to support their survival [23].

The reduction in catches of *G. tachinoides* was higher than that for *G. p. gambiensis* in both areas*.* This is consistent with observations from a study which conclusively demonstrated that *G. tachinoides* almost disappeared from the pastoral zone of Samorogouan (Burkina Faso) following the successful application of deltamethrin pour-on to cattle [22]. It is also easier to control *G. tachinoides* using insecticide-treated cattle since this fly species prefers cattle as hosts whereas *G. p. gambiensis* displays an opportunistic feeding behaviour (feeds on a wider range of hosts including monitor lizards) and is hence more difficult to control [22].

Previous experience has also shown that, unless there is implementation of a forward strategy (i.e. extend the area under control), any area is prone to reinvasion [13,24]. Otherwise, as in our case of an area about 500 km^2^ , the objective was limited to tsetse control rather than elimination.

Trypanosome prevalences in both areas were comparable before intervention. There was also a high prevalence of multiple drug-resistant *T. congolense* in both areas [9]. This changed following tsetse control resulting in an almost 8-fold risk reduction in the area under tsetse control compared to the area without intervention. This reduction is consistent with results from other tsetse control activities [12,24].

Tsetse control led to a risk reduction of contracting AAT, as expected. The share of infections with *T. congolense* was lower relative to that with *T. vivax*, which could also have been mechanically transmitted [25]. It is also acknowledged that young stock is particularly prone to infections with *T. vivax* as was shown in the Ghibe valley, Ethiopia [26].

Strongyles were predominant in Sikasso as was the case in other studies in West Africa [27-30]. Egg shedding was seasonal, decreasing during the dry season and then recovering during the rainy season. Egg output suppression occurs during the dry season since some nematode species (*Cooperia* species, *Bunostomum* species and *Oesophagostomum* species) survive as adults while others like *Haemonchus* species survive as inhibited larvae in the mucosa of the gastro-intestinal tract of their hosts [27,28].

We observed that egg shedding was dependent on certain animal-specific factors (results not presented here). For instance, cross-bred animals (zebu x trypanotolerant breeds) had comparatively lower FECs than zebu cattle [15], consistent with findings by Mattioli *et al*[31]. Additionally, young animals (up to 12 months of age) had higher egg shedding than older ones [15] indicating a build-up of immunity with increasing age [32].

It appears that AAT is better tolerated if helminth infections are treated. The risk of AAT in cattle treated with albendazole within the intervention area dropped nearly threefold (2.9 times) compared to the risk in cattle receiving a placebo, although this was not significantly different. A number of reasons could have caused the lack of an outright effect of albendazole treatment on trypanosome infections. Firstly, there was suspected nematode resistance to albendazole, limiting successful nematode control. An anthelmintic drug is considered effective against nematodes if the FECR in anthelmintic treated animals is 95 % and /or the lower bound of the 95 % confidence level must be at least 90 % [21]. In this study, neither of the two thresholds was attained indicating that the treatment was not fully effective in controlling gastro-intestinal nematodes. The causes for this phenomenon are not fully understood. Resistance against albendazole cannot be excluded although, it has not previously been described as a problem in the cotton zone of West Africa. It is also not known which strongyle species were not or insufficiently reacting to the anthelmintic treatment since larval cultures were not performed. Secondly, *refugia* could have diluted the effect of the albendazole treatments through continued re-infection [33,34]. Finally, inadequate blinding of the investigators could have made herd keepers aware about the treatments used leading to clandestine treatments with albendazole of cattle belonging to the placebo group.

Consistent with results of trypanosome prevalence surveys during intervention, the data at the end of the intervention indicated a significantly (p < 0.001) reduced trypanosome prevalence in the intervention area from 13.9 % before testing of best-bet strategies to 0.8 % after their implementation. However, a drop in the trypanosome prevalence in the control area was also observed, falling from 17.5 % before the study to 5.8 % at the end. This was attributed to simultaneous reduction of tsetse flies during the study period.

Before intervention multiple drug-resistant *T. congolense* were dominant in both areas [9], but no *T. congolense* were detected at the end of intervention in the area benefiting from tsetse control. This means that the tested integrated best-bet package greatly reduced *T. congolense* populations thereby contributing to the containment of trypanocidal drug resistance. Dominance of *T. congolense* had been persisting in the control area at comparable levels before the start of the trial.

Notwithstanding the effectiveness of the integrated best-bet strategies in containing trypanocide resistance, more research is required to demonstrate to what extent reversal of resistance is possible. Further studies to establish the economic viability of such integrated packages are warranted.

## Conclusions

Maintaining cattle productive in regions of high AAT risk is an elusive goal. The situation may be aggravated by the appearance of drug resistant trypanosome strains. Reliance on the strategic use of trypanocidal drugs alone is not a viable option. An integration of several strategies – combining use of trypanocides, tsetse control and measures aiming at better health of cattle at risk – is likely to be the only remaining viable option.

## Competing interests

The authors declare that they have no competing interests.

## Authors’ contributions

EOM carried out the field surveys, assembled data, analyzed and drafted the manuscript. OD, TFR, BB and PHC participated in study coordination, design and revised the manuscript. MPOB performed statistical analysis and revision of the manuscript. AH, YS and BM carried out field surveys and revised the manuscript. BH designed the helminth component of the study. KHZ reviewed the manuscript. All authors read and approved the final manuscript.
